# Image statistics substantiate Gaudí’s naturalistic design principles

**DOI:** 10.1038/s41598-025-06007-8

**Published:** 2025-06-20

**Authors:** Olga Dyakova, Karin Nordström, Christian Benedict

**Affiliations:** 1 Department of Medical Cell Biology, Uppsala University, 17 751 24, Uppsala, Sweden; 2 Flinders Health and Medical Research Institute, Flinders University, GPO Box 2100, Adelaide, SA 5001 Australia; 3 Department of Pharmaceutical Biosciences, Uppsala University, 751 24, Uppsala, Sweden

**Keywords:** Architecture, Buildings, Natural forms, Amplitude spectrum slope, Shannon entropy, Information technology, Environmental impact

## Abstract

Human observers perceive natural and man-made environments differently, a distinction measurable through image statistics. However, limited evidence exists on how architectural style influences these statistics and, consequently, visual perception. Understanding this relationship is essential, as architectural design shapes both our visual and psychological experiences of built environments. The amplitude spectrum slope quantifies sharpness and detail in an image, with values closer to 1 typically found in photographs of natural scenes. Image entropy, reflecting unpredictability, also plays a role in visual attention—images with higher entropy are more likely to capture interest. In this study, we analyzed photographs of buildings designed by Antoni Gaudí, renowned for his nature-inspired architecture. Our findings reveal that Gaudí’s buildings display an amplitude spectrum slope more similar to that of natural scenes than contemporary structures from the same area, alongside higher image entropy. Effect size measures indicated that the observed differences in slope constant and entropy between images of Gaudí buildings and contemporary buildings were medium and large in magnitude. The presence of trees in front of contemporary buildings shifts their image statistics toward naturalistic values. These results suggest that incorporating naturalistic design elements into architecture can alter image statistics, potentially influencing perception and aesthetic experience. In contemporary architecture, where minimalist and geometric styles are prevalent, these insights highlight the potential benefits of reintroducing complexity and naturalistic aesthetics to create more engaging and psychologically restorative built environments.

## Introduction

More than half of the global population currently resides in urban areas, and this number is still growing. Living in urban environments can strongly affect human mental health and well-being^1^. For example, images of natural environments are often associated with positive feelings, such as improved mood and restoration. In contrast, urban scenes may trigger negative emotions like stress and low mood^2^. In addition, design features like small spaces, cramped layouts, limited sunlight, poor ventilation, and lack of control over noise and other stressors can harm residents’ mental health^3,4^.

The processing of naturalistic input - how people see and respond to real-world images - can be studied using methods such as psychophysics^5^ and functional magnetic resonance imaging^6^. Additionally, image statistics help quantify the visual features of objects and scenes^7^. One important measure is the amplitude spectrum slope, which describes how light and dark areas are arranged at different levels of detail, from broad shapes to fine textures^8–15^. Interestingly, photos of natural scenes, often seen as highly pleasant^16–18^usually have an amplitude spectrum slope close to one^9,19,20^. Psychophysical research shows that the human visual system is especially sensitive to the amplitude spectra found in nature^21,22^. In contrast, scenes with slopes that differ from this natural range are often linked to higher stress^23^. By examining the amplitude spectrum slope of images of objects, we can understand how they might stand out in a scene and affect how people see them^24,25^.

Another key feature of visual environments is Shannon entropy, which measures how unpredictable an image is^26^. High entropy means the image has more variety and information, making it more interesting and likely to hold attention^15,26^. Amplitude spectrum slope and Shannon entropy together offer complementary ways to understand image structure, both of which help explain how visual environments affect us psychologically.

This study explored whether buildings designed with natural forms show an amplitude spectrum slope closer to 1 or higher Shannon entropy compared to modern, functional buildings. To test this, we analyzed photographs of three types of scenes: buildings by Antoni Gaudí (1852–1926), nearby contemporary buildings, and surrounding natural environments. Gaudí’s work often imitates natural forms, featuring unique window shapes, detailed roofs, and mathematical elements in his designs^27,28^. Biomimicry uses ideas from nature, such its shapes, systems, and processes, to create sustainable and effective design solutions. Gaudí’s architecture reflects this approach through organic shapes, nature-inspired structures, and a balance of form and function—principles that match modern biomimetic design.

We hypothesized that, because of its biomimetic nature, Gaudí’s architecture would have an amplitude spectrum slope more like natural scenes and show higher Shannon entropy than contemporary buildings (constructed post-2010). We also tested whether adding green spaces to photos of contemporary buildings would shift their amplitude spectrum slope closer to 1 and increase their entropy. By studying these differences, we aim to better understand how biomimetic architecture influences perception and psychological responses.

## Materials and methods

### Photographs

Photographs of the buildings were captured from a typical pedestrian’s viewing angle as they traversed down the street. In instances where the sidewalk was narrow, images were acquired from the opposite side of the road. Consequently, photographs without trees offered a ground-level perspective, encompassing details from the pavement to the roof. For images of buildings with trees, we adjusted our shooting distance based on the tree’s location. This broader framing included more of the surrounding space, incorporating the sky, thereby introducing variations in the image statistics. All photos were taken using a single-lens reflex digital Nikon D5600 camera, capturing images in RAW (.NEF) format at a resolution of 6000 × 4000 pixels. A fixed f/5 aperture was employed, allowing the camera to automatically adjust the shutter speed for optimal exposure. The ISO sensitivity was set at 160. The photography sessions occurred from late April to early May, 2023, between 10 a.m. and 3:15 p.m., during sunny weather with occasional passing clouds.

A total of 27 photographs of Gaudí’s buildings and 29 photographs of contemporary buildings were selected based on predefined criteria, including image resolution, unique parts of the buildings, perspective consistency, and the absence of obstructions (e.g., people or vehicles). The number of images was determined by the availability of high-quality photographs that met these standards.

### Gaudí’s buildings

**Fig. 1:**
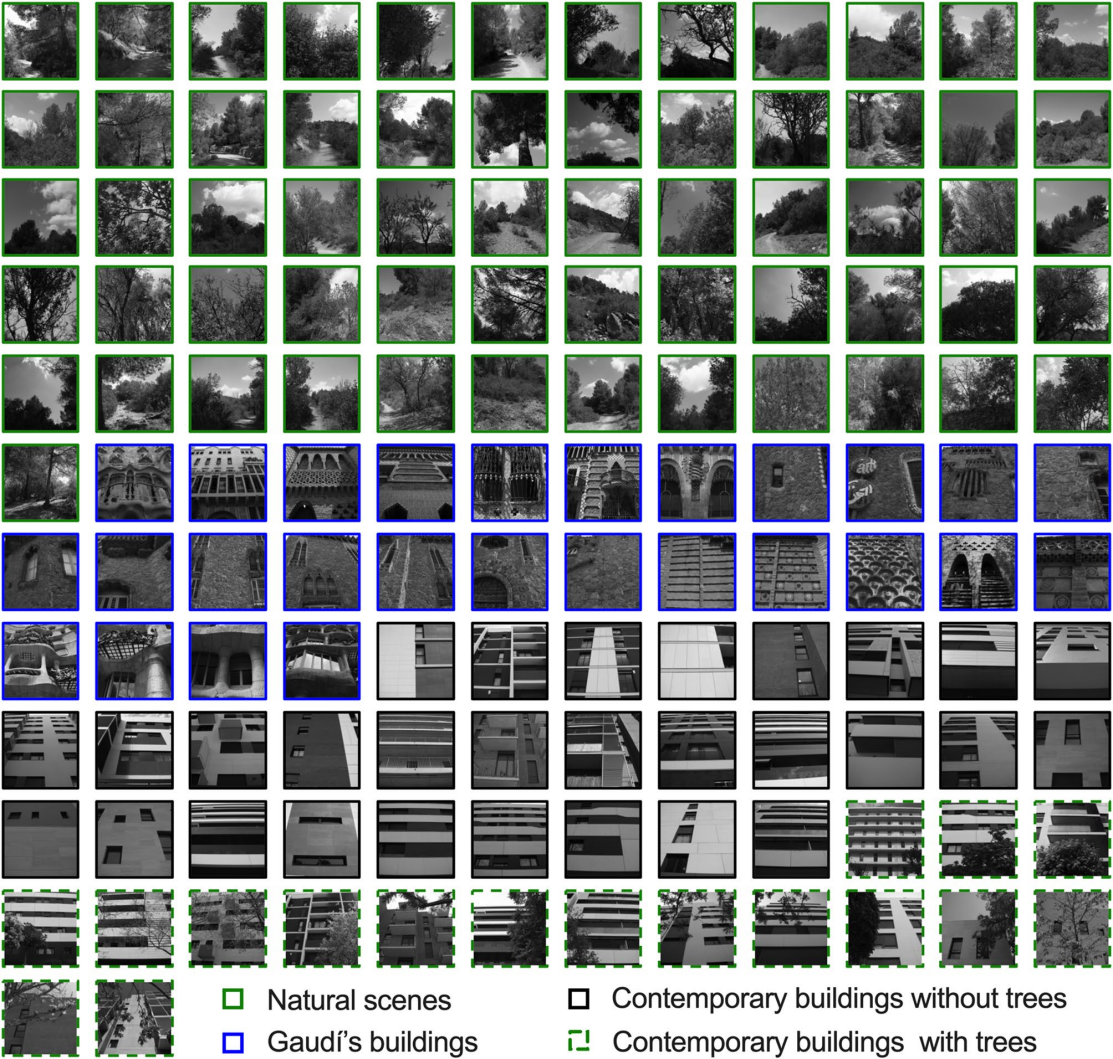
Collection of grayscale images for analysis. Green frames indicate images representing natural scenes, blue frames indicate Gaudí’s buildings, black frames indicate contemporary buildings, and dashed green frames represent contemporary buildings with trees in front of them.

We captured 27 photos of iconic Gaudí structures, including Güell Pavilions, Casa Vicens Gaudí (https://casavicens.org), Casa del Guarda in Park Güell (https://parkguell.barcelona), Palau Güell (https://inici.palauguell.cat), Bellesguard (https://bellesguardgaudi.com), Casa Milà (La Pedrera, https://www.lapedrera.com), and Casa Batlló (www.casabatllo.es). For details, see Fig. 1; Table 1.

**Table 1:** Geolocations of the buildings and natural scenes.

Image category	Geolocation
Natural scenes	
Collserola Natural Park	41°24’57.3”N 2°04’24.4"E
Gaudí’s buildings	
Casa Vicens Gaudí	41°24’13.0”N, 2°09’02.6"E
Casa Milà	41°23’43.6"N, 2°09’42.8"E
Casa Batlló	41°23’30.8"N, 2°09’53.7"E
Bellesguard	41°24’34.7"N, 2°07’36.6"E
Palau Güell	41°22’48.2"N, 2°10’27.1"E
Casa del Guarda in Park Güell	41°24’49.2"N, 2°09’13.1"E
Güell Pavilions	41°23’22.4"N, 2°07’09.8"E
Contemporary buildings	
Mas Lluí, Sant Feliu de Llobregat: 1 building	41°23’03.3"N 2°03’19.7”E
Roses-Castellbell, Sant Feliu de Llobregat: 4 buildings	41°22’56.2"N 2°03’18.5”E
Els Miralls, Sant Just Desvern: 2 buildings	41°22’46.3"N 2°03’32.2”E
Mas Lluí, Sant Just Desvern: 10 buildings	41°23’12.3"N 2°03’34.4"E

### Contemporary buildings

This set comprised 29 photographs of 17 buildings situated in 4 family-friendly neighborhoods within the Province of Barcelona, Spain (in the same region as Gaudí’s buildings). All these buildings were constructed post-2010 (Fig. 1). We chose contemporary buildings from the same area where Gaudí’s buildings stand for several key reasons. First, selecting buildings from the same geographical context allowed for a direct comparison between the visual characteristics of Gaudí’s architectural style and those of contemporary structures within the same urban environment. This approach enabled us to isolate architectural style as the primary variable influencing image statistics, minimizing potential confounding factors related to geographic or environmental influences. Additionally, choosing buildings from the same area provided a meaningful contrast between architectural styles while maintaining a consistent urban setting, enhancing the relevance of the comparison. Notably, each of these contemporary buildings featured trees in front of them, allowing us to capture 17 matched photographs with and without trees of the same structures. For details, see Fig. 1; Table 1.

### Natural scenes

We took 61 photos in Collserola Natural Park (the Province of Barcelona) to determine the distribution of image statistics of natural scenes and use these distributions as a reference for naturalness in our buildings’ analysis. For details, see Fig. 1.

### Image statistics

**Fig. 2:**
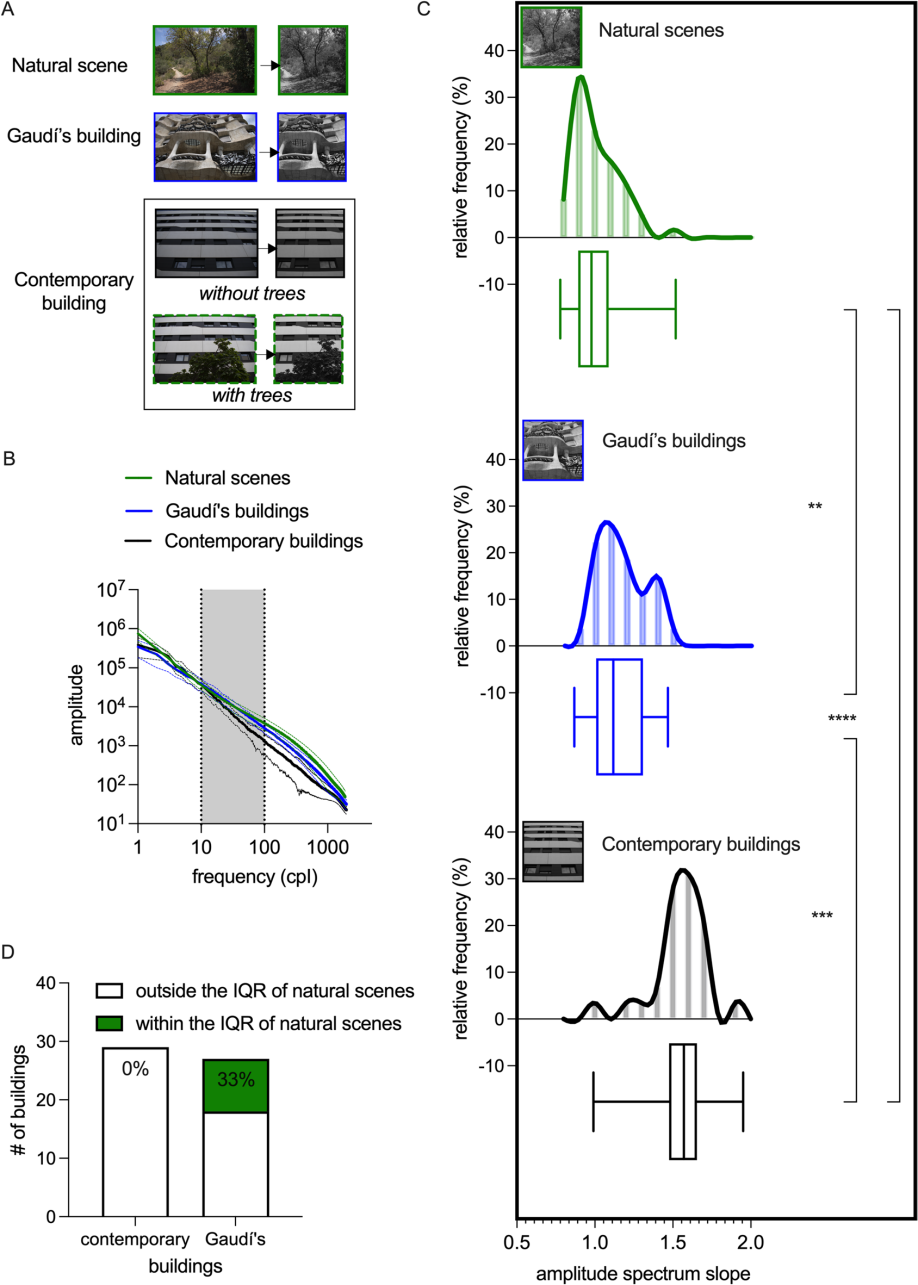
Amplitude spectrum slope of Gaudí’s buildings compared to contemporary architecture. (**A**) Example photographs used for analysis. Each original RGB photo (left column) was cropped into a 4000 × 4000 pixel square and converted to grayscale (right column). (**B**) Rotational averaged amplitude spectrum of photographs for natural scenes (green, *n* = 61), buildings designed by Gaudí (blue, *n* = 27), and contemporary buildings (black, *n* = 29). Thick lines depict the rotational average amplitude spectrum across all images for each category, with dashed thin lines representing standard deviation. The grey box highlights the spectrum part used for calculating the amplitude spectrum slope. (**C**) The amplitude spectrum slope of Gaudí’s building photos (blue) was significantly lower than that of contemporary buildings (black) and closer to the amplitude spectrum slope of natural scenes (green). ****p < 0.0001; ***p < 0.001; **p < 0.01. (**D**) 33% of Gaudí’s buildings’ images had an amplitude spectrum slope within the range of 1 and 1.2, 0% of contemporary buildings had an amplitude spectrum slope within this range.

For image statistics, we converted each .NEF photo without compression to .tif format images with a resolution of 300 dpi in Adobe Photoshop (https://www.adobe.com/). Then, we used Matlab R2022b (http://www.mathworks.com) to transform each photo into grayscale and then to double format using built-in functions *rgb2gray* and *im2double* correspondingly (for a more detailed description, see^9^). To minimize the presence of nearby buildings and the sky, we cropped the images to a central 4000 × 4000 pixel square (see Fig. 2A). This crop was designed to retain a high-resolution region that preserved the structural and statistical characteristics of the full image while being computationally manageable. The crop was centered, and equal amounts were extracted from both sides of the longer (horizontal) dimension to ensure the most relevant content was preserved. Image statistics were then extracted from this cropped region.

To calculate the average amplitude spectrum slope, we first performed a Fourier transform of the grey-scaled images (for details, see^9^). Then, we quantified the average amplitude across all orientations as a function of spatial frequency and plotted this on a log–log scale. We fit a linear function to this average amplitude spectrum ($\:\frac{1}{{f}^{{\upalpha\:}}}$) between 10 and 100 cycles per image (cpI) and extracted the amplitude spectrum slope (α), which was rounded to two decimals.

We calculated the Shannon entropy using Matlab’s function *entropy*, which is based on the following equation: $\:\text{E}\:=\:-\sum\:\text{p}\times\:{\text{l}\text{o}\text{g}}_{2}\left(\text{p}\right),\:$ (1)

where E is the Shannon entropy and p contains the normalized histogram counts returned from Matlab’s function *imhist*^29^. The extracted values were rounded to two decimal places. Higher Shannon entropy signifies increased randomness or complexity within the image, whereas lower Shannon entropy implies greater uniformity and predictability in the distribution of pixel values^15,26^. Note, the Shannon entropy takes into account only the probabilities of the intensity values and does not consider their spatial distribution^26,30^. For example, the pixels of white noise images have equal probabilities for each intensity and are uncorrelated within the image, so their entropy is maximum^12^.

### Statistical analyses

Data in the text are given as mean ± SD, unless otherwise specified. All statistical analyses were carried out using Prism versions 10.0.2 and 10.1.1 (GraphPad Software, USA, www.graphpad.com) and Microsoft Excel version 16.08 (Microsoft Corporation). We quantified the frequency distribution of image parameters for all images using the histogram function in Prism. Then we used a cubic spline to interpolate the data points. We applied D’Agostino and Pearson normality tests. As a result, we found that the amplitude spectrum slope of two of the three image categories (i.e., natural scenes and contemporary buildings) violated the assumption of normal distribution. Regarding entropy, the natural scene image category was not normally distributed. Consequently, we applied Kruskal-Wallis tests for both outcome measures. If a significant main effect of image category was demonstrated by Kruskal-Wallis testing (*p* < 0.05), post-hoc comparisons among the three image categories were performed by Dunn’s test with correction for multiple comparisons.

We computed effect sizes for both the Dunn’s and Wilcoxon signed-rank tests using the rank-biserial correlation^31^and for the parametric tests, we used Cohen’s d^32^. For the Dunn’s and Wilcoxon tests, the effect size was calculated as r = Z/√N, where Z is the standardized test statistic and N is the number of observations (or number of non-zero difference pairs in the Wilcoxon test). This provides an intuitive measure of effect size strength, with thresholds of 0.1, 0.3, and 0.5 corresponding to small, medium, and large effects, respectively^33^. For the t-test, Cohen’s d was calculated as the difference between group means divided by the pooled standard deviation, with conventional thresholds of 0.2, 0.5, and 0.8 indicating small, medium, and large effects, respectively. Considering that the average amplitude spectrum slopes of natural scenes are typically found to be between 1 and 1.2^9,12,15,34^, we applied a chi-square test to scrutinize any disparities between Gaudí’s and contemporary buildings in terms of the percentage of amplitude spectrum slopes within the range of ≥ 1.00 and ≤ 1.20. Additionally, to explore potential differences in amplitude spectrum slopes and entropy between contemporary buildings with and without trees, we employed either the Wilcoxon matched-pairs signed rank test (slope) or the Student’s matched-pairs t-test (entropy), depending on the results of D’Agostino and Pearson normality tests. Overall, a p-value smaller than 0.05 was considered significant.

In the figures, the central mark of each boxplot represents the median, the edges of the box denote the 25th to 75th percentiles, and the whiskers extend from the smallest to the largest values in the dataset.

## Results

### Amplitude spectrum slope

Rotational averaged amplitude spectrum is shown in Fig. 2B. A significant main effect of image category emerged (*p* < 0.0001, Kruskal-Wallis test, Fig. 2C). The mean amplitude spectrum slope of natural scenes was 1.01 ± 0.14, followed by Gaudí’s buildings (1.17 ± 0.17) and contemporary buildings (1.55 ± 0.18). The mean amplitude spectrum slope of Gaudí’s buildings was notably lower than that of contemporary buildings from the same area (*p* = 0.0001, Dunn’s test; rank-biserial effect size = 0.55). Furthermore, both building categories had a significantly higher slope than natural scenes (Gaudí’s buildings: *p* = 0.0038, rank-biserial effect size = 0.34; contemporary buildings: *p* < 0.0001, rank-biserial effect size = 0.86; Dunn’s test). 33% of Gaudí’s buildings’ images had an amplitude spectrum slope within the range of 1 and 1.2. In contrast, among images of contemporary buildings, none of the images fell within this slope range (*p* = 0.0007 for Gaudí’s vs. contemporary buildings, Chi-square test; Fig. 2D).

**Fig. 3:**
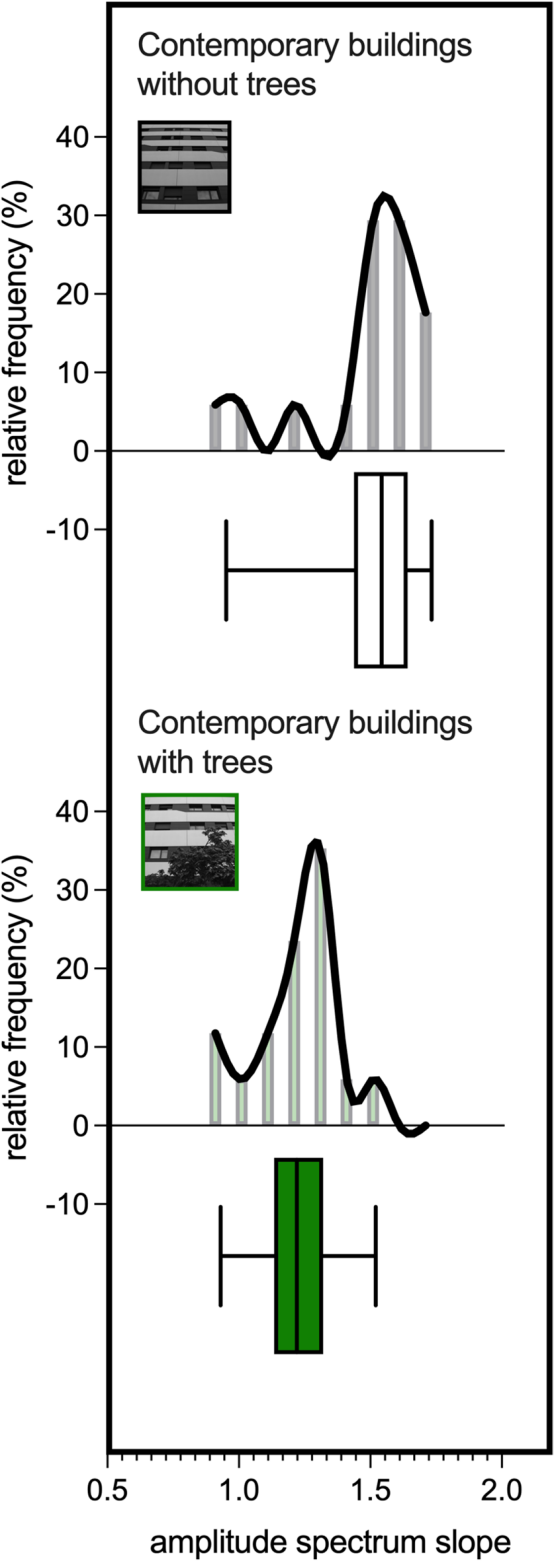
Amplitude spectrum slope of contemporary buildings with trees. The amplitude spectrum slope of contemporary buildings with trees in front of them (*n* = 17) was closer to one than when the buildings did not have trees (*n* = 17). ****p < 0.0001; ***p < 0.001; **p < 0.01.

When integrated with trees, the mean amplitude spectrum slope of photographs depicting contemporary buildings significantly decreased (from 1.50 ± 0.23 to 1.22 ± 0.16, *p* = 0.0045, Wilcoxon matched-pairs test; rank-biserial effect size = -0.66; Fig. 3). Consequently, 35% of the images of contemporary buildings with trees had an amplitude spectrum slope within the range of 1 and 1.2 (compared to 0% of the same buildings without trees; *p* = 0.007, Chi-square test).

### Shannon entropy

**Fig. 4:**
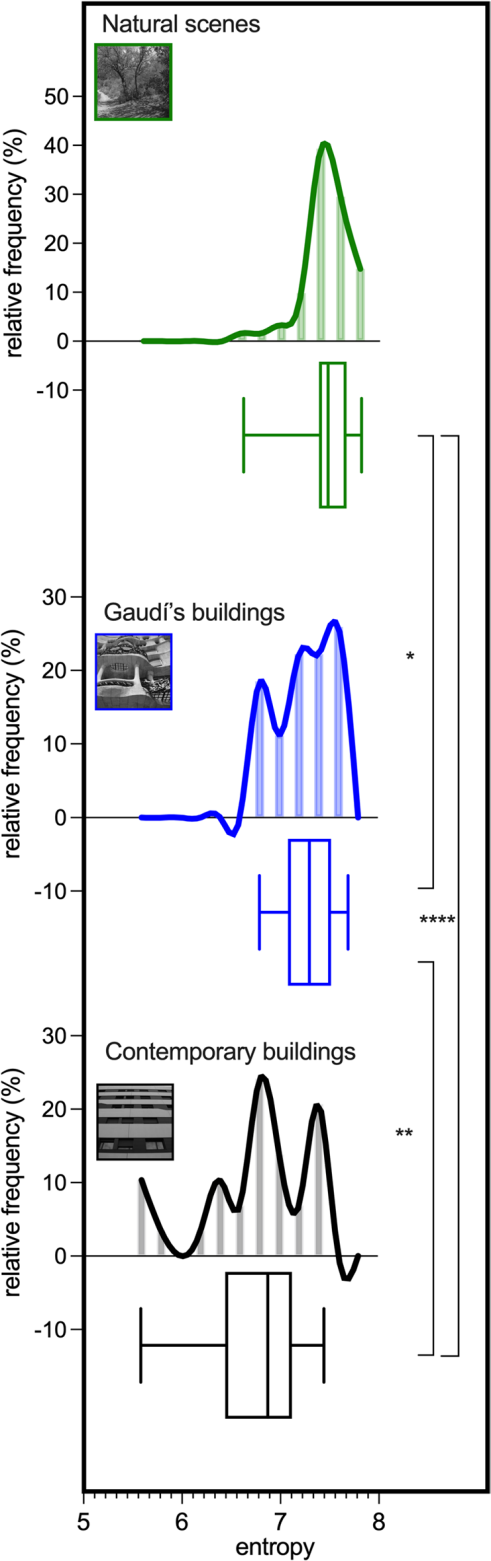
Shannon entropy in Gaudí’s buildings vs. contemporary structures. The mean Shannon entropy of Gaudí’s buildings’ photographs (blue, *n* = 27) was significantly higher than that of contemporary buildings (black, *n* = 29) and closer to the entropy of natural scenes (green, *n* = 61).

**Fig. 5:**
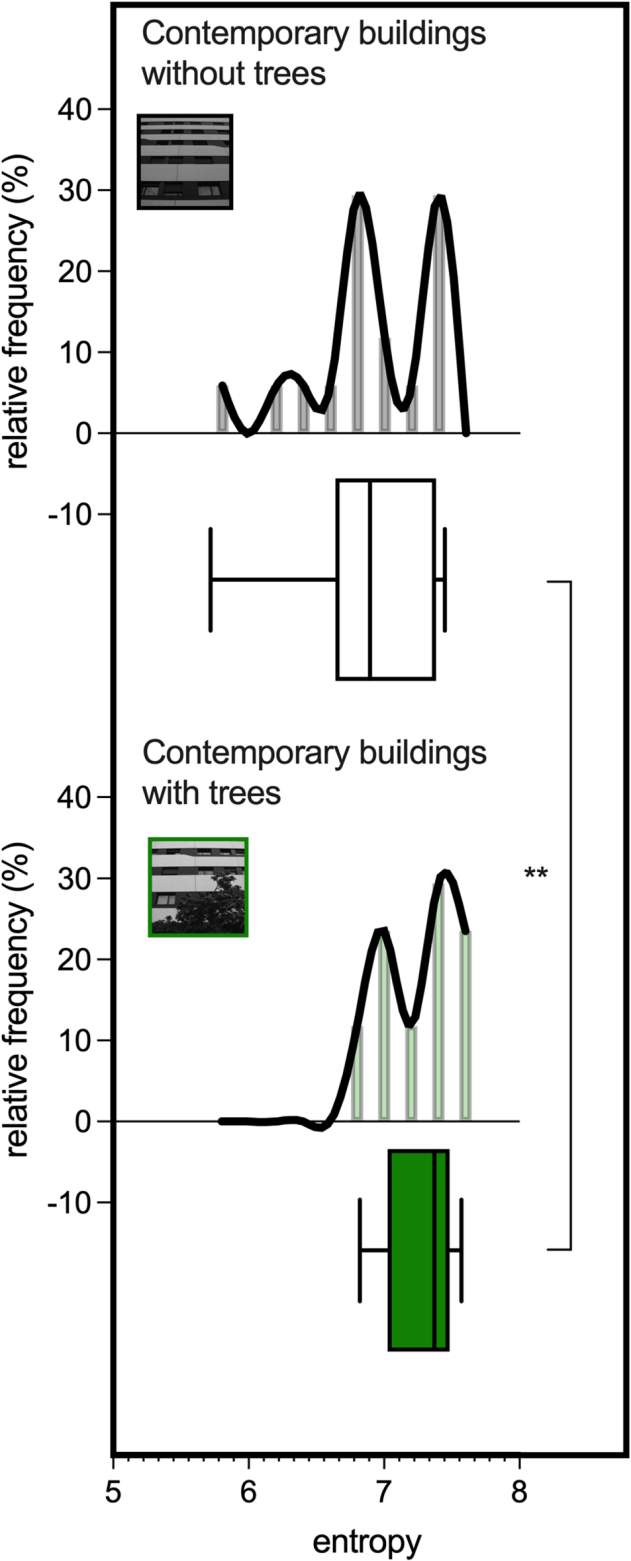
Shannon entropy in contemporary buildings with trees vs. contemporary buildings without trees. Shannon entropy in photos of contemporary buildings with trees was higher than in photos of the same buildings without trees (*n* = 17). ****p < 0.0001; **p < 0.01; *p < 0.05.

As indicated by the Kruskal-Wallis test, a main effect of image category on mean entropy was observed (*p* < 0.0001, Fig. 4). The highest mean entropy was identified for natural scenes (7.47 ± 0.23), followed by Gaudí’s buildings (7.28 ± 0.27; *p* = 0.014 vs. natural scenes, rank-biserial effect size = 0.30; *p* = 0.004 vs. contemporary buildings, rank-biserial effect size = 0.43; Dunn’s test). The lowest mean entropy was recorded for contemporary buildings (6.75 ± 0.56; Dunn’s test; *p* < 0.0001 vs. naturalistic scenes; rank-biserial effect size = 0.71). Notably, the entropy of contemporary buildings with trees was 5.4% significantly higher than that of the same buildings without trees (7.27 ± 0.25 vs. 6.90 ± 0.48, *p* = 0.0016, Student’s matched-pairs t-test, Cohen’s d = 0.92; Fig. 5).

## Discussion

In this study, we found that the average amplitude spectrum slope, which measures the distribution of light and dark areas across spatial frequencies in an image^8^of Gaudí’s buildings in Catalonia was closer to that of natural scenes than buildings constructed after 2010. Specifically, 33% of Gaudí’s building photos had an amplitude spectrum slope between 1 and 1.2, typical for natural scenes^8,19,35^. In contrast, no photos of contemporary buildings showed slopes in this range, suggesting that Gaudí’s nature-inspired designs align more closely with the visual characteristics of nature than recent functional architecture.

To explore the visual complexity of these buildings, we calculated Shannon entropy, which measures the variety within an image’s pixel intensity^30,36^. Gaudí’s buildings had higher entropy, indicating greater visual complexity. The use of materials like rough stone and colorful ceramics, along with intricate decorations like carvings and mosaics, added both tactile and visual richness, contributing to unpredictability. Gaudí’s nature-inspired designs, which deviated from traditional norms, further increased the visual complexity. An analysis of the greyscale images showed that this unpredictability comes from the combination of materials, textures, and unconventional forms. People are naturally drawn to areas with higher entropy, as they offer more visual information^36,37^. The higher entropy, along with a higher amplitude spectrum slope, suggests that Gaudí’s buildings are more visually engaging, potentially enhancing well-being in urban spaces.

In addition to analyzing Gaudí’s buildings, we also examined how green spaces affect the visual properties of contemporary architecture. Exposure to natural environments or viewing natural scenes is known to elicit positive emotional responses^38–43^which has driven interest in designing green buildings that support both sustainability and well-being. Our analysis showed that photos of contemporary buildings with nearby trees had amplitude spectrum slopes closer to one, a value typical of natural scenes^16^. This suggests that incorporating green spaces may enhance the visual appeal of architecture.

### Limitations

However, several limitations should be considered. First, our analysis focused solely on buildings in Catalonia, Spain, which may limit the generalizability of the results to other regions or cultures. Additionally, although we examined how architectural style and green spaces affect amplitude spectrum slope, we did not assess whether these differences influence subjective ratings of aesthetic appeal. Prior research has shown that natural scenes with steeper amplitude spectrum slopes are rated as less pleasant under certain conditions, such as sleep deprivation^44^. Based on this, we hypothesize that Gaudí’s buildings, whose amplitude spectrum slopes are closer to one, may be perceived as more aesthetically appealing, though this remains to be tested.

Another limitation is that while we identified differences in amplitude spectrum slope and entropy across architectural styles, these effects may not hold in larger or more diverse image sets. Future studies with broader datasets could help validate and expand upon our findings. Moreover, our study focused on visual image statistics and did not account for other sensory dimensions, such as acoustics or tactile features, which contribute to architectural experience. The connection between visual aesthetics, well-being, and image statistics is likely shaped by a range of psychological, cultural, historical, and individual factors. To deepen our understanding, future work should integrate subjective evaluations and explore the role of additional sensory cues in shaping responses to the built environment.

## Conclusions

Our findings suggest that architecture inspired by natural forms can be more visually captivating and engaging, offering a promising way to improve the built environment. However, to fully understand its impact, further psychological research is needed to establish a clear link between these visual features and emotional outcomes, such as reduced stress and enhanced well-being. Our study also indicates that contemporary architectural styles, which often diverge from natural form principles, could benefit from incorporating elements that reflect nature, such as greenery and organic patterns, to boost their visual appeal. While practical challenges remain, including maintenance costs, limited urban space, and concerns about long-term sustainability, our research highlights the important role of architecture and urban planning in creating environments that not only look appealing but also support emotional well-being. By adopting biomimetic principles, cities can develop spaces that are both aesthetically pleasing and beneficial to mental health.

## Data Availability

The data and statistical codes are publicly available at 10.5061/dryad.qfttdz0qf.
